# Tolerance of Spermatogonia to Oxidative Stress Is Due to High Levels of Zn and Cu/Zn Superoxide Dismutase

**DOI:** 10.1371/journal.pone.0016938

**Published:** 2011-02-18

**Authors:** Fritzie T. Celino, Sonoko Yamaguchi, Chiemi Miura, Takashi Ohta, Yuzuru Tozawa, Toshiharu Iwai, Takeshi Miura

**Affiliations:** 1 Research Group for Reproductive Physiology, South Ehime Fisheries Research Center, Ehime University, Ainan, Ehime, Japan; 2 Cell-Free Science and Technology Research Center, Ehime University, Matsuyama, Ehime, Japan; University of Queensland, Australia

## Abstract

**Background:**

Spermatogonia are highly tolerant to reactive oxygen species (ROS) attack while advanced-stage germ cells such as spermatozoa are much more susceptible, but the precise reason for this variation in ROS tolerance remains unknown.

**Methodology/Principal Findings:**

Using the Japanese eel testicular culture system that enables a complete spermatogenesis *in vitro*, we report that advanced-stage germ cells undergo intense apoptosis and exhibit strong signal for 8-hydroxy-2′-deoxyguanosine, an oxidative DNA damage marker, upon exposure to hypoxanthine-generated ROS while spermatogonia remain unaltered. Activity assay of antioxidant enzyme, superoxide dismutase (SOD) and Western blot analysis using an anti-Copper/Zinc (Cu/Zn) SOD antibody showed a high SOD activity and Cu/Zn SOD protein concentration during early spermatogenesis. Immunohistochemistry showed a strong expression for Cu/Zn SOD in spermatogonia but weak expression in advanced-stage germ cells. Zn deficiency reduced activity of the recombinant eel Cu/Zn SOD protein. Cu/Zn SOD siRNA decreased Cu/Zn SOD expression in spermatogonia and led to increased oxidative damage.

**Conclusions/Significance:**

These data indicate that the presence of high levels of Cu/Zn SOD and Zn render spermatogonia resistant to ROS, and consequently protected from oxidative stress. These findings provide the biochemical basis for the high tolerance of spermatogonia to oxidative stress.

## Introduction

Reactive oxygen species (ROS) are dangerously reactive molecules or free radicals (chemical species with an unpaired electron) generated as a byproduct of normal aerobic metabolism by the reduction of oxygen [1], [2]. They are constantly produced in normal cellular metabolism in all living organisms and excessively in pro-oxidant states. ROS expression at appropriate levels has physiological roles in cellular differentiation [3] and sperm capacitation [4] while oxygen-free radicals at concentrations beyond physiological limits result in oxidative stress. Oxidative stress is a disturbance in the balance between the production of ROS and antioxidant defenses, which can damage DNA, proteins, and lipids [5], [6], ultimately leading to apoptosis or necrosis in living cells [7]. However, there are only few reports examining the direct impact of ROS on germ cells.

Germ cells are highly specialized cells that are responsible for the propagation of DNA which directs development of future generations. To ensure the continuation of the species, it is essential for organisms to maintain the integrity of germ cell DNA. Previous studies have shown that spermatogenic cells have lower mutation frequency than somatic cells [8], [9]. Yet, germ cells are continually affected detrimentally by endogenous and exogenous agents such as ROS that can cause DNA damage. Spermatozoa were found to be highly sensitive to ROS-induced damage [10], while spermatogonia are reportedly tolerant to ROS [11]. Previous studies revealed that in mice exposed to mild heat stress, which can consequently lead to oxidative stress [12], numerous apoptotic late-type germ cells were found while apoptotic spermatogonia were rare [13]. However, the precise reason for this phenomenon remains to be clarified. In vertebrates, a variety of antioxidant defense mechanisms have evolved to protect cells and tissues against ROS. Among the well known antioxidant enzymes protecting cells from ROS are the superoxide dismutases (SOD). SODs specifically metabolize oxygen-free radicals and are believed to be the first and one of the most important lines of antioxidant enzyme defense systems against ROS. Superoxide (O_2_^•−^) and hydroxyl (OH^•−^) radicals generated from free oxygen in cells [14] are the primary and the most active ROS containing unpaired electrons making them highly reactive to biomolecules [15]. SODs specifically scavenge superoxide anion radicals and convert them into hydrogen peroxide and oxygen which in turn are broken down into water by catalase in peroxisomes, and glutathione peroxidase in the cytosol and mitochondria [16]. Three distinct types of SODs have been identified in mammals. Two isoforms have copper (Cu) and zinc (Zn) in their catalytic center and are located either in the cytoplasm (Cu/Zn SOD) or in the extracellular elements (EC SOD). The third isoform has manganese (Mn SOD) as a cofactor and is located in the mitochondria [17]. SODs catalyze the dismutation of the superoxide anion radical to oxygen and hydrogen peroxide [18]. Among SOD isotypes, Cu/Zn SOD activity has been found to be higher in seminal plasma in mammals [19]. In Cu/Zn SOD, both Zn^2+^ and Cu^2+^ are needed to thermally stabilize the native enzyme and maintain full catalytic activity. However, Zn is considered less critical and can be substituted with cadmium, mercury or cobalt ions [20] while no other metal can replace copper in restoring catalytic function. On the other hand, in rats, dietary Zn deficiency induced an increase of lipid peroxidation in the liver, brain, and testes which may correlate to increased production of reactive oxygen species [21], and hence, an impaired antioxidant system. Zn deficiency-related diseases in humans, such as Crohn's disease and nutritional disorders, demonstrated an inhibition of spermatogenesis and sperm abnormalities [22], [23]. Although both Zn and Cu are important in reproduction in males and females, concentrations of Zn is high in the adult testis, and the prostate has the highest concentration of Zn in the body [22]. Zn plays a key regulatory and structural role in every mammalian cell [24]. Recently, we have shown that Zn deficiency through Zn chelation suppressed sperm motility and induces germ cell apoptosis in Japanese eel (*Anguilla japonica*) [25]. However, the precise mechanism underlying Zn function in protecting testis and/or gem cells has not been determined.

Under aquaculture conditions, the male Japanese eel has an immature testis containing only non-proliferating type A spermatogonia. However, a single injection of human chorionic gonadotropin (hCG) can stimulate the complete pathway of spermatogenesis from spermatogonial proliferation through spermiogenesis, wherein all late-type germ cells such as spermatocytes, spermatids and spermatozoa can be observed after 18 days of hCG injection [26]. Moreover, a testicular organ culture system for the Japanese eel has been developed in our laboratory, which is the only currently available system in vertebrates in which the induction of complete spermatogenesis can be performed *in vitro* by hCG or 11-ketotestosterone (11-KT, a fish-specific androgen derived from testosterone) stimulation [27]. Various factors which play important roles in fish spermatogenesis have been identified using this method [28], [29]. In the present study, we again utilized the Japanese eel model to examine the effects of ROS on germ cells and clarify the protective roles of Cu/Zn SOD and Zn in spermatogonia against ROS attack. The results demonstrated that Cu/Zn SOD is strongly expressed in spermatogonial stages but to a lesser extent in advanced-stage germ cells. We show that Zn levels decrease during spermatogenesis and Zn modulates the activity of SOD. Furthermore, we also show that knockdown of Cu/Zn SOD increased sensitivity of spermatogonia to exposure to hypoxanthine-generated ROS.

## Results

### Advanced-stage germ cells undergo apoptosis upon exposure to Hx-induced ROS while spermatogonia remain unaltered

**Figure 1:**
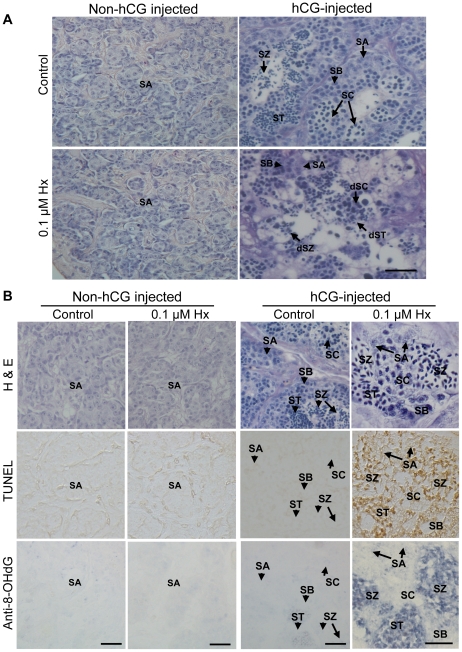
Hypoxanthine (Hx) induced apoptosis in advanced-stage germ cells but not in spermatogonia. (A) Representative light micrographs of non-hCG injected and hCG-injected Japanese eel testicular fragments cultured for 6 days with or without 0.1 µM Hx stained with H&E (hematoxyline-eosin). (B) TUNEL assay and immunohistochemistry for 8-OHdG in non-hCG injected and hCG-injected eel testicular fragments cultured for 3 days with 0.1 µM Hx. Positive signals for TUNEL assay and 8-OHdG are indicated by brown and blue staining, respectively. H & E, hematoxyline and eosin; SA, type A spermatogonia; SB, type B spermatogonia; SC, spermatocytes; ST, spermatids; SZ, spermatozoa; dSC, dead SC; dST, dead ST; dSZ, dead SZ. Bars, 20 µm.

To clarify the influence of ROS on spermatogenesis, we investigated the effects of ROS generated by the hypoxanthine/xanthine oxidase (Hx/XO) system on germ cells using testis at the initial stage of spermatogenesis in which only type A spermatogonia are observed, and hCG-injected eel testis containing germ cells at different stages such as, spermatogonia, spermatocyte, spermatids and spermatozoa [26]. Prior to this experiment, we detected XO activity in Japanese eel testis (Figure S1), which could be considered enough to generate free radicals based on a previous study [30], hence we used only Hx for generation of ROS. Six days after the start of culture, testicular fragments of the initial and control group showed normal histological structure and were occupied by spermatogonia, spermatocytes, spermatids, and spermatozoa. After culturing with Hx for 6 days, late germ cell stages such as spermatids and spermatozoa underwent significant cell death while spermatogonia remain unaffected (Figure 1A).

Figure 1B shows the results of TdT-mediated dUTP nick-end labeling (TUNEL) assay, an assay to detect apoptosis, and 8-hydroxy-2′-deoxyguanosine (8-OHdG) immunohistochemistry, a method to evaluate oxidative DNA damage. After 3 days of culture, control sections in both the initial control and hCG-injected eel testicular fragments did not have any apoptotic germ cells. However, germ cells at advanced stages such as spermatocytes, spermatid and spermatozoa were found to undergo intense apoptosis after Hx treatment. Spermatogonia did not exhibit signal for TUNEL while almost all spermatids and spermatozoa exhibited very strong signals for TUNEL.

To determine the extent of oxidative damage in germ cells, we examined the 8-OHdG signals in testicular fragments. There was no observed oxidative DNA damage in the control testicular fragments in both the non-hCG injected and hCG-treated groups after 3 days of culture. However, similar to results from the TUNEL assay, 8-OHdG immunohistochemistry showed that after Hx treatment, spermatocytes, spermatid and spermatozoa but not spermatogonia undergo oxidative DNA damage. Hx-treated sections of non-hCG treated group containing only type A spermatogonia did not show any positive signal for TUNEL and 8-OHdG. Although we examined the effects of various doses of Hx, we did not observe major differences between doses.

### Total SOD activity and Cu/Zn SOD protein level is high at early stages of spermatogenesis and in spermatogonia

**Figure 2:**
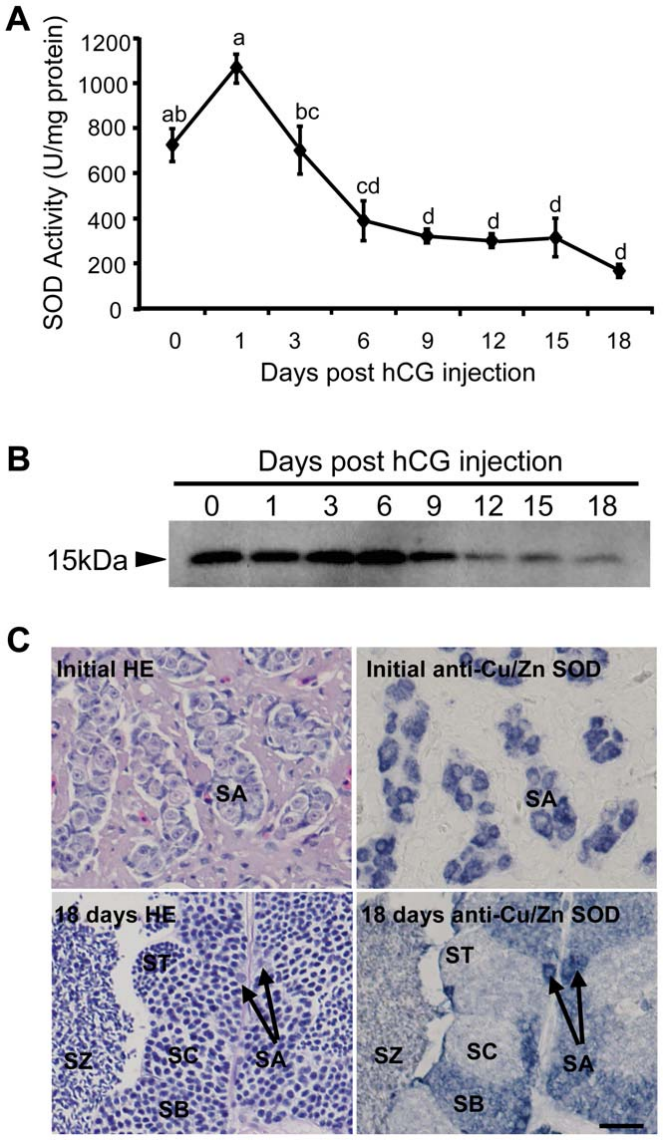
SOD activity and Cu/Zn SOD level decreases during spermatogenesis, and spermatogonia strongly expressed Cu/Zn SOD. (A) Total SOD activity assay in testis of eels (n = 5 per group) at various days of post-hCG injection. Values with different letters are significantly different (*P*<0.05). Values are expressed as mean ± SEM. (B) Western blot analysis using commercial mammalian anti-Cu/Zn SOD antibody in testis of Japanese eel after various days of hCG injection. (C) Immunohistochemistry for Cu/Zn SOD in testicular fragments of non-hCG injected (Initial) and 18 days post-hCG injected groups (18 days). SA, type A spermatogonia; SB, type B spermatogonia; SC, spermatocytes; ST, spermatids; SZ, spermatozoa. Bars, 20 µm.

Since Cu/Zn SOD has been considered the primary antioxidant defense in cells, we checked the SOD activity and changes in Cu/Zn SOD protein expression in testis of eels at various days of post hCG-injection. SOD activity assays showed a high activity at day 0 and at 1 day after hCG injection wherein only type A and early type B spermatogonia (resting spermatogonia before the initiation of spermatogenesis) can be observed, but a decreasing activity was observed as spermatogenesis progressed after 1 day through 18 days post-hCG injection as advanced germ cells appeared: late type B spermatogonia at day 3 to 6, spermatocytes at day 12, and spermatids and spermatozoa at day 18 (Figure 2A).

To examine the expression of Cu/Zn SOD protein in the testis in the course of spermatogenesis, we conducted Western blot analysis using a mammalian anti-Cu/Zn SOD antibody in testis of eels collected after various days of hCG injection. A band of 15 kDa for eCu/Zn SOD was successfully detected that gradually increased in density until 6 days post-hCG injection when the testis was occupied mainly by spermatogonia, and then progressively decreased in density until 18 days post-hCG injection (Figure 2B).

To determine the distribution of Cu/Zn SOD in different germ cell stages of testis, we conducted immunohistochemistry on eel testis at initial stages of spermatogenesis wherein only type A spermatogonia are present and 18 days post-hCG injection when all germ cell stages are present. Strong expression of Cu/Zn SOD was observed in type A and B spermatogonia, but expression was weak in more advanced germ cells such as spermatocytes, spermatids, and spermatozoa (Figure 2C). These results imply differences in the antioxidant system among spermatogenic cells.

### Zn level is higher in spermatogonia and lower in late-type germ cells

**Figure 3:**
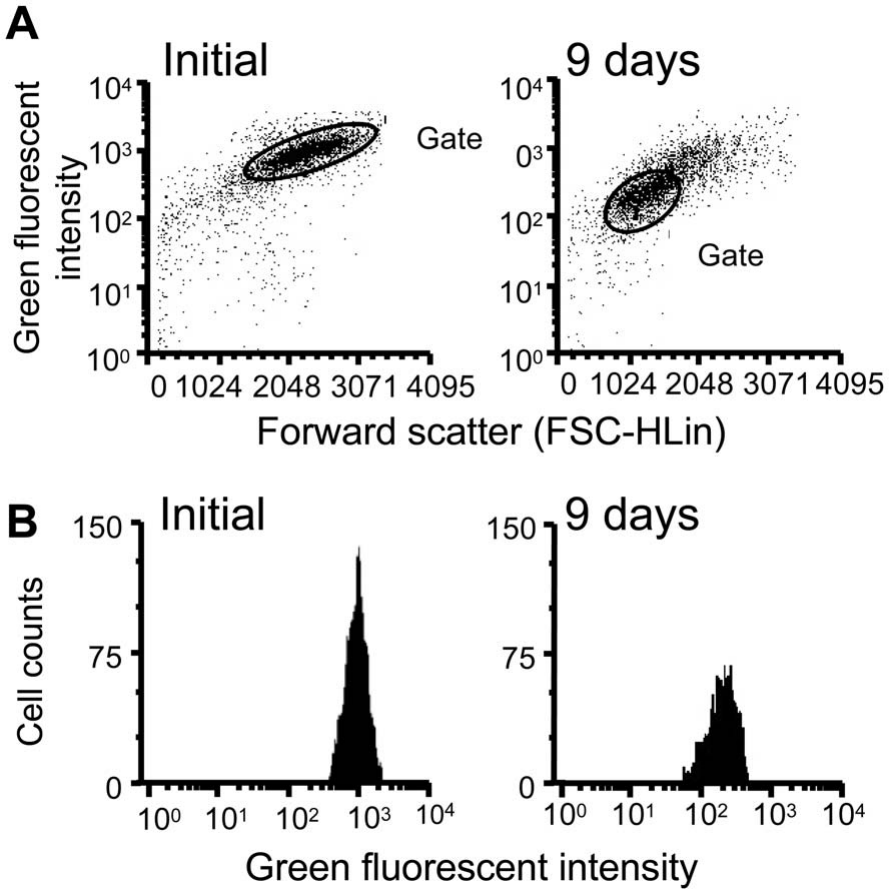
Spermatogonial stem cells contain higher levels of Zn than late-type germ cells. Dot plot of gated germ cells (A). (B) Histograms of germ cells of non-hCG injected (Initial) and 9 days post-hCG injected (9 days) eels.

Previously, we reported that Zn is present at high levels in germ cells in testis and chelation of Zn causes germ cell apoptosis [25]. Therefore, to determine the possible protective role of Zn against oxidative stress in germ cells, we analyzed the Zn distribution in germ cells using flow cytometry. Among initial control and hCG-treated eels, different distributions and strength of Zn fluorescence were observed. In the initial control, almost all germ cells belong to large size group consisting only of type A spermatogonia. These germ cells exhibited high intensity of Zn fluorescence (Figure 3A). Nine days after hCG injection in eel, spermatogenesis progressed until just before the start of meiosis, and the testis was occupied mainly by late type B spermatogonia as shown in our previous studies [28], [31]. In these testes, the germ cell size became smaller, and the intensity of Zn fluorescence decreased 5x less than the Zn fluorescence intensity mean of the initial control (Figure 3A and B, Table 1). These results suggest that Zn concentration in germ cells decreases as they developed into advanced stage.

**Table 1:** Flow cytometric analysis of Zn levels in germ cells.

	Ratio of cells in gate (%)	Fluorescence means	SEM
Initial	85.99	1019.82	6.79
Day 9	61.00	240.6	5.16

### Zn modulates eel testicular Cu/Zn SOD activity

We cloned the eel *Cu/Zn SOD* (*eCu/Zn SOD*) from the testis in order to synthesize recombinant eel Cu/Zn SOD (re-eCu/Zn SOD) protein. To clone the full-length cDNA of this clone, a complete cDNA library was screened and a positive clone of 828 bp in length was obtained. The putative protein consisted of 154 amino acids (Figure S2, GenBank Acc. No. AB558958). The residues involved in coordinating Cu (His-47, 49, 64, 121) and Zn (His-64, 72, 81 and Asp-84); and the Arg144 which is considered an important residue for Cu/Zn SOD enzyme activity [32]; and two cysteine residues at positions 58 and 147, which form an intra-chain disulfide bridge, are all conserved in the *eCu/Zn SOD*. Database searches showed that the deduced amino acid sequence of this clone is highly homologous to teleost and mammalian Cu/Zn SOD (Figure S3A). Phylogenetic relationship analysis showed that the predicted amino acid sequence of eCu/Zn SOD is highly related to cytosolic Cu/Zn SOD of other species, rather than to other types of SOD (Mn SOD and EC SOD) (Figure S3B), confirming that the cDNA sequence encodes a bona fide cytosolic Cu/Zn SOD.

**Figure 4:**
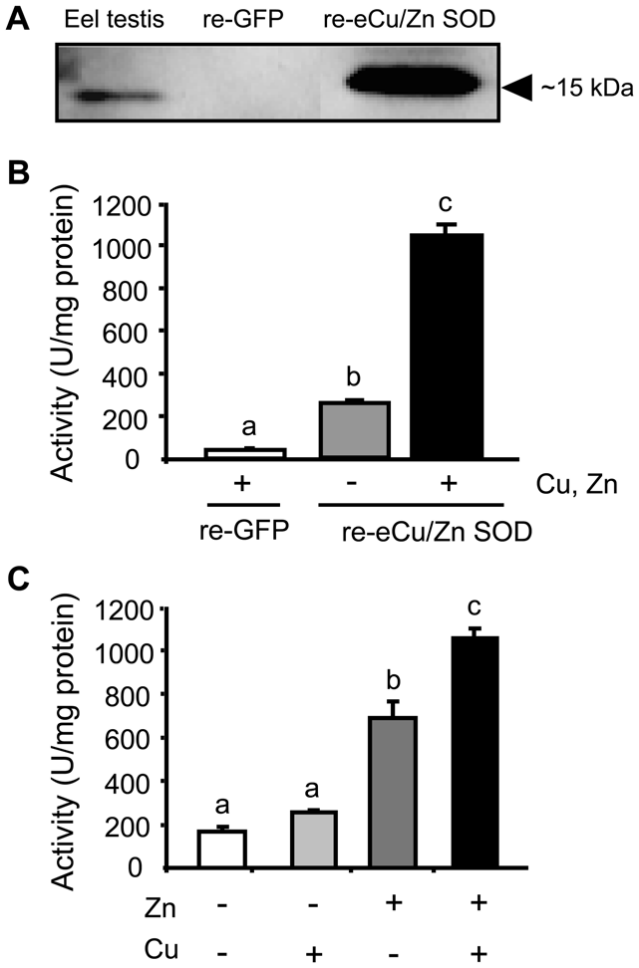
Deficiency in Zn reduces Cu/Zn SOD activity. (A) Western blot analysis of eel testis homogenate and recombinant eel Cu/Zn SOD (re-eCu/Zn SOD) using a commercial mammalian anti-Cu/Zn SOD. Recombinant green fluorescent protein (re-GFP) was used as a negative control. (B) Enzymatic activity assay of re-eCu/Zn SOD with or without addition of 10 µM of Cu (CuSO_4_) and 100 µM Zn (ZnCl_2_) (n = 3 per group). (C) Effects of Zn on the activity of re-eCu/Zn SOD. The activity of recombinant protein with or without addition of either 10 µM of Cu or 100 µM Zn or both was assayed (n = 3 per group). -, without Cu and Zn; +, added with Cu and Zn. Values with different letters are significantly different (*P*<0.05). All data are represented as mean ± SEM.

Using this clone, we synthesized a His-tag re-eCu/Zn SOD using a wheat germ cell-free protein synthesis system to confirm that the *eCu/Zn SOD* gene obtained from Japanese eel in this study is indeed the eel testis *eCu/Zn SOD*, and retains its catalytic function by Western blot analysis and SOD activity assay. Western blot analysis of the purified translation mixture containing re-eCu/Zn SOD using a mammalian anti-Cu/Zn SOD antibody revealed a band with a molecular mass of 15 kDa which was similar to the predicted molecular weight (15.4 kDa) of the eCu/Zn SOD protein and detected a similar size protein in eel testis homogenate. No band was detected in recombinant green fluorescent protein (re-GFP) (Figure 4A). Moreover, after addition of 10 µM and 100 µM final concentrations of Cu and Zn respectively, which are roughly equal to the levels observed in gonads of freshwater fish [25], [33], the re-eCu/Zn SOD translation mixture was observed to have a very high enzymatic activity compared to the mixture not added with ions (*P*<0.01). The negative control of the mixture containing translated re-GFP, has a very low activity compared to both the re-eCu/Zn SOD with or without Cu and Zn (*P*<0.01, Figure 4B). These results indicate that a functional eCu/Zn SOD enzyme was obtained from Japanese eel testis.

Afterwards, we examined the effects of Zn on the activity of the re-eCu/Zn SOD to confirm the importance of Zn in the antioxidant response in germ cells. We found that the addition of Zn alone increased the enzymatic activity of the re-eCu/Zn SOD to a level higher than that of the negative control and Cu alone addition, and increased the enzymatic activity just slightly lower to that of the Zn and Cu added to re-Cu/Zn SOD (Figure 4C). These results imply that Zn affects the activity of the eCu/Zn SOD.

### Short interfering RNA (siRNA) transfection decreased Cu/Zn SOD-expressing spermatogonia and increased oxidative damage and cell death

**Figure 5:**
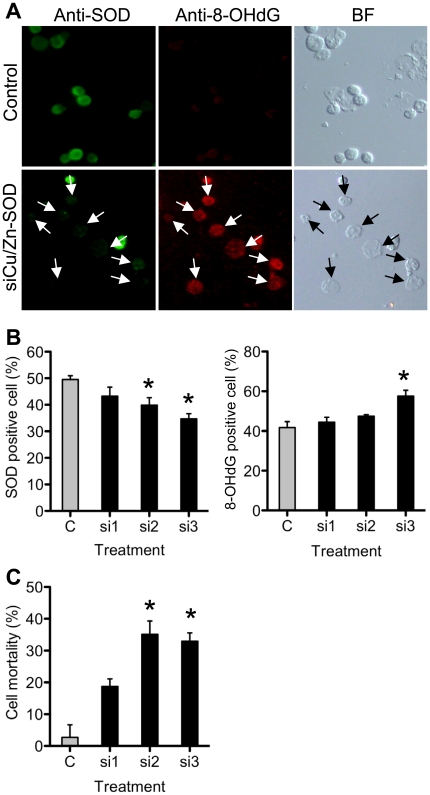
Cu/Zn siRNA decreased Cu/Zn SOD-positive spermatogonia and increased oxidative damage and mortality. (A) Photomicrographs of fluorescence immunohistochemistry of siRNA transfected spermatogonia after 3 days of culture. Green staining indicates Cu/Zn SOD positive signal, red staining indicates 8-OHdG positive signal. Arrows indicate Cu/Zn SOD-negative cells in Cu/Zn SOD immunohistochemitry or 8-OHdG-positive cells in 8-OHdG immunohistochemistry. BF, bright field. (B) Calculated number of spermatogonia expressing Cu/Zn SOD and positive for 8-OHdG after siRNA transfection (n = 5 per group, *P*<0.05). The percentage of these cells was calculated as compared to control. (C) Mortality of spermatogonia exposed for 6 days to Cu/Zn SOD siRNA (n = 5; *P*<0.01). *, significantly different from control. si1, si2, s13; siRNA1, 2, and 3, respectively. Values are expressed as mean ± SEM.

To further confirm the importance of Cu/Zn SOD in the maintenance of spermatogonia, we used the cloned *eCu/Zn SOD* to generate *eCu/Zn SOD* siRNA for loss of function experiments and to investigate the effects of siRNA oligonucleotide duplex on spermatogonia survival. Transfection efficiency of siRNA into isolated spermatogonia was 35.9 ± 2.32%. After siRNA knockdown, the expression of Cu/Zn SOD and synthesis of 8-OHdG was investigated using immunohistochemistry. Immunohistochemistry was done after 3 days of transfection since this is an optimum period for observation and analysis of gene/protein expressions and oxidative DNA damage. After transfection of three different siRNA in germ cells, Cu/Zn SOD-positive germ cells decreased, while the number of 8-OHdG-positive cells increased when compared to siRNA control (Figure 5A and B). Transfection of siRNA 3 in which the target site is located at open reading frame (ORF) region of the *eCu/ZnSOD* was most effective in reducing Cu/Zn SOD expression and increasing 8-OHdG synthesis in germ cells. Also, it was observed that almost all cells strongly expressing Cu/Zn SOD either exhibit weak or no signal for 8-OHdG. Dead germ cells were counted after 6 days, a period wherein cell death was manifested. Treatment of siRNA for 6 days caused approximately 15% and 30% cell mortality in siRNA1, and siRNA 2 and 3, respectively (Figure 5C), suggesting that siRNA-transfected spermatogonia died.

## Discussion

Although effects of ROS have been extensively studied in mammals not much is known about its direct impact on vertebrate germ cells. In this current report we present a solid basis for the differences in ROS sensitivity in germ cells, providing the underlying reason for the high tolerance of spermatogonia to ROS.

ROS at minimum levels was found to have a variety of physiological roles such as in sperm capacitation [4], antimicrobial defense, cell signaling [34], and cell differentiation [3]. However, enhanced generation of ROS has detrimental effects on cells, and can compromise fertilizing capacity and genetic integrity of spermatozoa [10], [35]. ROS up-regulation can also cause profound changes in gene expression and can lead to oxidative damage in cells [36]. Frequent exposure of humans to ionizing radiation may cause male infertility [37] and excessive ROS is associated with radiation-induced damage to germ line [38]. Nevertheless, the direct effects of ROS on germ cells are unclarified. By definition, ROS is often used by biologists to refer to oxygen radicals (O_2_^•−^), hydroxyl (OH^•−^), peroxyl (R O_2_^•−^) and alkoxyl (ROH^•−^) and certain nonradicals that are either oxidizing agents and/or are easily converted into radicals e.g., singlet oxygen (O_2_) and H_2_O_2_ [39]. In this study, we refer ROS to superoxide anion. Here, we examined the effects of ROS on various germ cell stages, using testis of immature and artificially matured hCG-injected eels. After Hx treatment, only advanced germ cell stages, most notably spermatids and spermatozoa, but not spermatogonia exhibited apoptosis concomitant with severe oxidative damage. These results indicate that later stage germ cells are less tolerant to ROS than early type germ cells. In previous studies in mice oxidatively stressed by selenium deficiency [40], spermatocytes, spermatids and spermatozoa were found in reduced number. Moreover, many studies in mammals indicated that spermatozoa are very susceptible to oxidative stress due to high concentrations of polyunsaturated fatty acids in the plasma membrane [41] and their limited store of antioxidant enzymes [35]. Spermatids were also found to be more sensitive to heat-induced stress compared to spermatogonia [13]. However, almost all of these studies focus on clarification on sperm sensitivity and their findings were unconfirmed. Furthermore, a detailed explanation of how sensitivities to ROS vary between germ cells, and the underlying reason for the high resistance of spermatogonia to ROS have yet to be determined.

Recently, we have shown that Zn is present at high levels in germ cells, and that chelation-induced Zn deficiency in testis resulted to apoptosis of germ cells of eels [25]. A number of studies suggested that a major result of Zn deficiency can be an increase in oxidative damage of tissues [42]. Thus, we hypothesized that Zn may be important to the antioxidant system response in germ cells. To confirm this hypothesis, we checked the distribution of Zn during spermatogenesis. We found that at early spermatogenesis, wherein most of the cells are spermatogonia, a high concentration of Zn was observed. The level of Zn was found to decrease as spermatogenesis progressed. This suggests differences in Zn levels in different germ cell types.

 Zn deficiency is known to result in hypogonadism, inhibition of spermatogenesis, and defects in the morphology of spermatozoa [43]. In addition, severe Zn deficiency leads to necrosis of germ-cell precursors that may lead to tubular atrophy, and abnormal differentiation of spermatids [44]. However, the precise mechanism by which Zn provides a protective role is unknown. It was suggested that Zn may be involved in several components of the oxidant defense including Cu/Zn SOD, an essential component of the antioxidant system [45]. To determine the underlying mechanism of Zn involvement as a cofactor of Cu/Zn SOD in the testicular antioxidant system and elucidate the antioxidant response of germ cells, we began examining the SOD activity, Cu/Zn SOD protein expression and distribution among germ cells.

 Cells are normally protected against oxidative damage by multiple enzymatic mechanisms and by antioxidant molecules. The SODs are the first and most important line of antioxidant enzyme defense systems against ROS and particularly superoxide anion radicals. In rats, it has been demonstrated that SOD activity in the testis varies with maturation [46]. Similarly, our study revealed a decreasing amount of total SOD activity as spermatogenesis progresses. In this study we focused on Cu/Zn SOD, since previous studies indicate that Cu/Zn SOD activity in testis is higher than other isoenzymes and that Cu/Zn SOD is the first enzyme involved in scavenging oxygen radicals [15], [19]. We found that the Cu/Zn SOD protein was highly expressed during the first few days of post-hCG injection. Moreover, among the germ cells, Cu/Zn SOD protein was highly expressed in spermatogonia. In agreement with the present results, in human testis, Cu/Zn SOD protein expression was shown to be intense in spermatogonia of the seminiferous tubules, while in spermatocytes and in other more differentiated germ cells and Sertoli cells, a weak expression was observed [47]. Moreover, in rat, a homogenous expression of Cu/Zn SOD mRNA was found in immature testis, while as maturation progresses, a heterogenous pattern of expression of Cu/Zn SOD mRNA was found with a depletion of mRNA in more mature cells [48]. These results may imply a role for Cu/Zn SOD in the protective response of spermatogonia.

Also, knockdown of Cu/Zn SOD resulted in a reduction of Cu/Zn SOD-expressing germ cells, an increase in 8-OHdG-positive cells, and increased cell mortality. Thus, a deficiency of Cu/Zn SOD appears to be related to the high vulnerability of spermatogenic cells to oxidative stress. In Cu/Zn SOD-knockout mice exposed to heat stress, cleavage of DNA in spermatogenic cells was found to be elevated [49]. Our experimental data demonstrates that Cu/Zn SOD is indeed a major factor in the high tolerance of spermatogonia to ROS.

Furthermore, it was shown that addition of Zn increased the enzymatic activity of re-eCu/Zn SOD. These results imply that Zn modulates the Cu/Zn SOD activity, and the deficiency of Zn reduces Cu/Zn SOD activity. Hence, this is the first study to demonstrate an important role for Zn as a cofactor of Cu/Zn SOD in the antioxidant system of spermatogonia.

Overall, our study demonstrated that the decrease or change in level of SOD activity and expression of Cu/Zn SOD, as well as of levels of Zn, as spermatogenesis progresses are the reasons for the vulnerability of advanced stage germ cells to ROS attack. The high levels of Cu/Zn SOD and Zn in spermatogonia may render them less susceptible to ROS attack. Since spermatogonia are on the unprotected side of the testis barrier, they may be prone to more DNA damage from circulating molecular insults than are cells on the protected side. As stem cells, spermatogonia need to ensure the integrity of genes required for development and the continuity of life. Thus, spermatogonia have evolved with a greater need for elevated levels of protective factors against DNA damage. These data provide a basis for directing future studies in spermatogenesis and antioxidant system in germ cells.

## Materials and Methods

### Ethics Statement

All experiments were conducted in strict accordance with the institutional animal ethics guidelines of Ehime University and were approved by the Animal Experimental Committee of Ehime University (Protocol Number 128, Permit Number H19-001).

### Animals

Cultivated male Japanese eel, (BW: 180–200 g) were purchased from a commercial eel supplier and kept in circulating freshwater tanks at 23°C until use. A single injection of hCG dissolved in eel Ringer's solution (146 mM NaCl, 3.07 mM KCl, 5.05 mM CaCl_2_, 4 mM MgCl_2_, 10 mM HEPES, 0.1% glucose, adjusted to pH 7.4) was intramuscularly administered at a dose of 5 IU/g BW after the fish were anaesthesized with 0.05% (v/v) Ethyl-p-Amino benzoate. Fish were sacrificed either immediately or at 1, 3, 6, 9, 12, 15, and 18 days after hCG injection which induces complete spermatogenesis within 18 days. The testes were then collected for activity assays, Western blotting, histological analysis, and immunohistochemistry experiments as described below.

### Testicular organ culture techniques

Five fish per group were anaesthetized with 0.05% (v/v) Ethyl-p-Amino benzoate before directly dissecting or before a single injection of hCG dissolved in eel Ringer's solution at a dose of 5 IU/g body weight, which induced complete spermatogenesis from spermatogonial proliferation to spermiogenesis within 18 days. Fish were sacrificed either immediately or after 12 days of post-hCG injection and the testes were collected. Freshly removed testes were cut into 1 mm×1 mm×0.5 mm pieces and placed on 1.5% (w/v) agarose (Agarose S, Wako Inc.) cylinders covered with a nitrocellulose membrane in 24-well plastic tissue culture dishes. Testicular fragments (n = 2–4) from each fish were randomly assigned to each control and treatment group and then cultured in 1 ml of Leibovitz' L-15 medium (Invitrogen, Ltd.) containing 10 mM HEPES, 1.7 mM L-proline, 0.1 mM L-aspartic acid, 0.1 mM L-glutamic acid, 0.5% (w/v) bovine serum albumin (BSA), and 1 mg/l bovine insulin with 0, 0.1, 1 and 10 µM hypoxanthine (Hx, Sigma-Aldrich) for 3 or 6 days at 20°C in humidified air. Testicular fragments were then fixed in Bouin's solution, embedded in paraffin wax and cut into 4 µm serial sections. The sections were stained with Delafield's hematoxyline-eosin for histological analysis.

### Analysis of germ cell death

To detect apoptosis, serial sections of 3-day-cultured testicular fragments from five fish of each group of non-hCG injected (initial control group) and artificially matured, hCG-injected eels analyzed for histology were used for TUNEL assay by a commercial kit (In Situ Cell Death Detection Kit, POD, Roche Diagnostics) following the manufacturer's protocol.

### Immunohistochemistry for 8-OHdG

8-OHdG immunohistochemistry using anti-8-OHdG monoclonal antibody (Japan Institute for the Control of Aging) was performed on serial sections of testicular tissue used for TUNEL to investigate oxidative DNA damage in testis following the manufacturer's protocol with some modifications. After deparaffinization and rehydration, the sections were microwave heated in 10 mM citric acid buffer (pH 6.0). The sections were then blocked in 1% (w/v) DIG blocking reagent (Roche Diagnostics) in phosphate buffered saline (pH 7.4) for 30 minutes at room temperature and incubated with primary antibody at 4°C overnight. Biotin-labeled rabbit anti-mouse IgG was used as the secondary antibody, followed by a streptavidin-alkaline phosphatase complex (Nichirei Biosciences Inc.).

### SOD enzymatic activity analysis in testis

To measure the SOD activity in testis, 100 mg of testis from 0, 1, 3, 6, 9, 12, 15, and 18 days post-hCG injected eels were homogenized in sucrose buffer. After adjusting the concentration of protein to 1 µg/µl, SOD activity assay was performed using a commercial competitive inhibition assay kit with a tetrazolium salt for detection of superoxide radicals generated by xanthine oxidase and xanthine (SOD Assay Kit-WST, Dojindo) following the manufacturer's protocol. Forty microliters of the resultant solution was used to prepare the sample according to the kit's protocol. One unit of SOD activity is defined as the amount of protein that inhibited tetrazolium reduction to 50% of maximum. The results were expressed as U/mg protein.

### SDS-Page and Western Blot analysis

We checked whether the commercial mammalian anti-Cu/Zn SOD antibody can react with eel Cu/Zn SOD in testis by Western blot analysis using the recombinant eel Cu/Zn SOD protein we have synthesized by cell-free protein synthesis system [50] and found that this antibody specifically detect eCu/Zn SOD in fish testis. Using this antibody, we conducted Western blot analysis on testis at various days of post-hCG injection to check the Cu/Zn SOD protein levels in testis during the course of spermatogenesis.

In brief, fresh testicular tissue explants from five eels in various days of post-hCG injection were homogenized in eel Ringer's solution at 4°C. The homogenates were mixed with sample buffer and boiled at 95°C for 5 min. Ten microgram of protein was resolved on 15% SDS-PAGE, and transferred to polyvinylidene difluoride membranes (Millipore). The membranes were then blocked with 5% skimmed milk in 20 mM Tris-HCl (pH 7.5) containing 0.5 M NaCl (TBS). After blocking, the membranes were then incubated with primary antibody against Cu/Zn SOD (1∶1000; Stressgen, Bioreagents Corp.) in 5% skimmed milk in TBS at 4°C overnight, washed, and incubated with a 1∶2000 dilution of alkaline phosphatase-conjugated anti-rabbit secondary antibody (Vector Laboratories) in 0.1% BSA in TBS for 1 hour at room temperature. After incubation of the membrane with CDP Star, bands were visualized using LAS-1000 mini (Fujifilm, Japan).

### Immunohistochemistry for Cu/Zn SOD

Immunohistochemistry for Cu/Zn SOD was performed using the commercial mammalian anti-Cu/Zn SOD antibody used for Western blot analysis. The testicular fragments from the initial (non-hCG injected) and 18 days post-hCG injected eels were fixed in Bouin's solution, embedded in paraffin and cut into 4 µm serial sections. The sections were deparaffinized in xylene and hydrated in a graded ethanol series. Immunohistochemical analysis was performed by using Histofine SAB-AP kit (Nichirei Biosciences) according to the manufacturer's protocol.

### Zinc concentration and distribution among germ cells during spermatogenesis

Flow cytometric assay was performed to determine the Zn distribution among germ cells. Samples were prepared according to previously described method [51]. Eels were injected with hCG to induce spermatogenesis. Immediately before or 9 days after injection of hCG, testes were collected, placed in ice-cold eel Ringer's solution, and minced by scissors and forceps. Testicular cells were isolated by collagenase and dispase. After treatment with DNase I, testicular cells were filtrated by two stainless meshes of decreasing pore size (75 and 45 µm), incubated in collagen-coated culture dish at 20°C overnight. After overnight incubation, only fibroblast, Sertoli and interstitial cells adhered to the bottom of collagen coated-culture dish. Germ cells were then moved into non-treated dish and were kept until use for flow cytometry.

Isolated germ cells were stained by Zn-specific fluorescent probe, ZnAF-2DA (Sekisui Medical Co., Ltd.) according to ref. 22. Briefly, isolated germ cells were washed 3 times by eel Ringer's solution, and incubated with 5 µM of the permeable Zn specific probe, ZnAF-2DA in eel Ringer's solution for 45 min at 20°C. Thereafter, stained germ cells were washed by eel Ringer's solution, treated with DNase I (Roche Diagnostics) for 15 min at 25°C, and filtrated by 20 µm-stainless steel mesh. Before flow cytometric analysis, germ cells were stained by a final concentration of 2 µg/ml of Propidium Iodide (PI) to exclude dead cells (PI positive cells) from analysis. Analysis was done by Guava EasyCyte Mini system (Millipore).

### Screening and cloning of full length eel *Cu/Zn SOD* cDNA

The cDNA clone of eel Cu/Zn SOD (*eCu/Zn SOD*) was obtained by using a SMART cDNA library construction kit (Clontech). The positive clone obtained from the library screening was sequenced by the dideoxy chain termination method using the Dual CyDye Terminator sequence kit (Amersham Biosciences) and a Long-Read Tower DNA sequencer (Amersham Biosciences). The homology search of the deduced amino acid sequence of the obtained cDNA was carried out using the FASTA in the DNA Data Bank of Japan web site (http://www.ddbj.nig.ac.jp/search/fasta-j.html). Multiple-sequence alignments and phylogenetic trees were generated by using the CLUSTALW software (Figure S3).

### Cell-free protein synthesis

We synthesized a recombinant eCu/Zn SOD to check whether the cDNA obtained is indeed the real Cu/Zn SOD from testis and to be utilized for investigation of Zn effects on Cu/Zn SOD activity in testis.

The N-terminus 8 his-tagged gene specific forward primer with *Spe*I site, 5′ ACTAGTATGCATCACCATCACCATCACCATCACGCACTGAAGGCAGTT-3′ and the gene specific reverse primer with *Sal*I site 5′-GTCGACTTACTGCGTGATGCCAAT-3′ were used to amplify the cDNA fragment encoding the ORF of *eCu/Zn SOD* by RT-PCR. The amplified fragments were then subcloned into a pGEM-T Easy vector and the fragment was checked for correct size and purity. The resulting product containing the full-length open reading frame was digested with *Spe*I and *Sal*I, and the digested fragments were subcloned into pEU3b expression vector [52]. mRNA was prepared by *in vitro* transcription with SP6 RNA polymerase (Promega), and a bilayer cell-free protein synthesis was performed with wheat germ extract as previously reported [53]. The resulting His-tag recombinant eel Cu/Zn SOD protein (re-eCu/Zn SOD) was then purified by TALON Metal Affinity Resin (Clontech Laboratories Inc.). For confirmation of the obtained protein, Western blot analysis was performed as described above.

### Enzymatic activity analysis of re-eCu/Zn SOD

Activity analysis of recombinant eCu/Zn SOD, re-eCu/Zn SOD, was performed using a commercial kit as described above. The translation mixture containing recombinant green fluorescent protein (re-GFP) used as a negative control, and re-eCu/Zn SOD which was produced with wheat germ cell-free protein synthesis system were buffer exchanged into sucrose buffer (0.25 M sucrose, 10 mM HEPES and 1 mM EDTA, pH 7.4) using Microspin G25 column (GE Healthcare Biosciences). Forty microliters of the resultant solution was then added with Cu (CuSO_4_) and Zn (ZnCl_2_) to a final concentration of 10 µM and 100 µM, respectively, to express the protein and the sample solution was then prepared for activity assay. The concentrations of Cu and Zn were based on previous studies [22], [30].

To analyze the effect of Zn ions on the activity of the re-eCu/Zn SOD, 0.8 mg of protein was assayed with or without 10 µM Cu alone or 100 µM Zn alone or both Cu and Zn. The sample solution was then prepared and the assay was performed as described above.

### Silencing of the eCu/Zn-SOD expression in germ cells using siRNA

To evaluate the role of eCu/Zn-SOD in germ cells *in vitro*, *eCu/Zn SOD* siRNA was used to reduce the expression of eCu/Zn-SOD levels in spermatogonia. Three pairs of double stranded siRNA oligonucleotides for eCu/Zn-SOD were synthesized by Hayashi Kasei Co., Ltd., and designated as siRNA1, 2, and 3. For the siRNA sequences and positions of target sites, please refer to Table S1 and Figure S2. Briefly, 15 pmol of *eCu/Zn-SOD* or control siRNA (BLOCK-iT™Alexa Flour® Red Fluorescent Oligo, Invitrogen) were diluted in 50 µl Opti-MEM I Reduced Serum Medium (Invitrogen) and then mixed. Lipofectamine RNAiMAX (1 µl) was diluted in 50 µl Opti-MEM I Reduced Serum Medium, mixed in diluted siRNA solution, and incubated for 15 min to form the siRNA/Lipofectamine RNAiMAX complex. Thereafter, 500 µl of cell suspension (5×10^4^ cell) from each fish (n = 5) in L-15 [23] were mixed into siRNA/Lipofectamine RNAiMAX complex. siRNA-applied germ cells were incubated at 20°C for 3 or 6 days. After 3 days of culture, a period in which gene/protein expression and DNA damage can be clearly observed, germ cells were attached into slide glasses for analysis of synthesis of 8-OHdG and expression of eel Cu/Zn-SOD by immunohistochemistry. After 6 days of culture, the phase in which cell death can already be observed, the germ cell numbers were counted using Trypan blue stain for the evaluation of cell mortality.

For immunohistochemistry, after germ cells were attached to slides and slides were dried at room temperature, the slides were washed three times by PBS then microwave heated in 10 mM citric acid buffer (pH 6.0). After washing with PBS and PBS-Tween twice, the slides were blocked in 1% (w/v) DIG blocking reagent (Roche Diagnostics) in phosphate buffered saline (pH 7.4) for 45 minutes at room temperature and incubated with primary antibodies for 1 hour at room temperature. After incubation, the slides were washed thrice by PBS and incubated with Alexa Flour 488 anti-rabbit IgG or Alexa Flour 555 anti-mouse IgG (Invitrogen) for 8-OHdG and Cu/Zn SOD, respectively, for 1 hour at room temperature. The slides were then washed with PBS and PBS-Tween twice and mounted in 50% glycerol.

### Statistical Analysis

All values were expressed as mean ± standard error of means (S.E.M). A one-way analysis of variance followed by Tukey's multicomparison test was used to analyze the differences of means using KaleidaGraph statistical software.

## Supporting Information

Figure S1 **Xanthine oxidase (XO) activity can be detected in testis.** Assay for XO was performed on testis of eels (n = 5 per group) at various days of post-hCG injection.
(TIF)

Figure S2 ***eCu/Zn SOD*** **nucleotide sequence and siRNA target sites.** Eel *Cu/Zn SOD* (*eCu/Zn SOD*) gene was obtained by cDNA library screening in eel testis. Gray shaded region indicates ORF region. Bold letters are deduced amino acids sequence. The three target sequences for the siRNA are underlined. *, stop codon.
(TIF)

Figure S3 **Deduced amino acid sequence, sequence alignment and phylogenetic analysis of** ***eCu/Zn SOD*** **.** (A) Deduced amino acid sequence of *eCu/Zn SOD* and alignment with other species Cu/Zn SOD. Two cysteine residues are indicated by arrows. The copper (Cu) binding sites are indicated by asterisk and the zinc (Zn) binding sites are indicated by † sign. The common binding sites for Cu and Zn are indicated by an open circle. Dots are identical amino acids among the proteins. The eCu/Zn SOD amino acids sequence is highly homologous to salmon, *Salmo salar* (AY736282); zebrafish, *Danio raerio* (Y12236); grouper, *Epinephelus coides* (AY735008) (77.7–80.8%); human, *Homo sapiens* (AY450286) and mouse, *Mus musculus* (M35725) (67.6–71.7%), Cu/Zn SOD proteins. (B) Phylogenetic analysis of SOD proteins. Sequences were aligned using CLUSTAL W. Gene bank accession nos. for amino acid sequences are: chimpanzee (*Pan troglodytes*) Mn SOD (AB087274), mouse Mn SOD (X04972), zebrafish Mn SOD (AY195857), pufferfish, *Takifugu obscurus*, Mn SOD (EF667049), zebrafish Cu/Zn SOD, salmon CuZn SOD, grouper Cu/Zn SOD, catshark, *Scyliorhinus torazame*, Cu/Zn SOD (DQ988331), human Cu/Zn SOD, mouse Cu/Zn SOD, African frog, *Xenopus laevis*, Cu/Zn SOD (BC070696), roundworm, *Caenorhabditis elegans*, EC SOD (AB190513), chicken, *Gallus gallus*, EC SOD (BX929804), mouse EC SOD (AF223251), salmon EC SOD (BT046917) and zebrafish EC SOD (CT737206). Branch lengths indicate proportionality to the amino acid changes on the branch. Scale bar shows substitution per site. Mn SOD, manganese SOD; EC SOD, extracellular SOD.
(TIF)

Table S1 Cu/Zn-SOD siRNA targeting sequences and Cu/Zn-SOD siRNA sequences. S, sense; A, antisense.
(DOC)
