# Supplementary figures and images for: Tolerance of Spermatogonia to Oxidative Stress Is Due to High Levels of Zn and Cu/Zn Superoxide Dismutase
Source: PLoS One. 2011 Feb 18;6(2):e16938. doi: 10.1371/journal.pone.0016938 (PMC3041797; doi:10.1371/journal.pone.0016938)

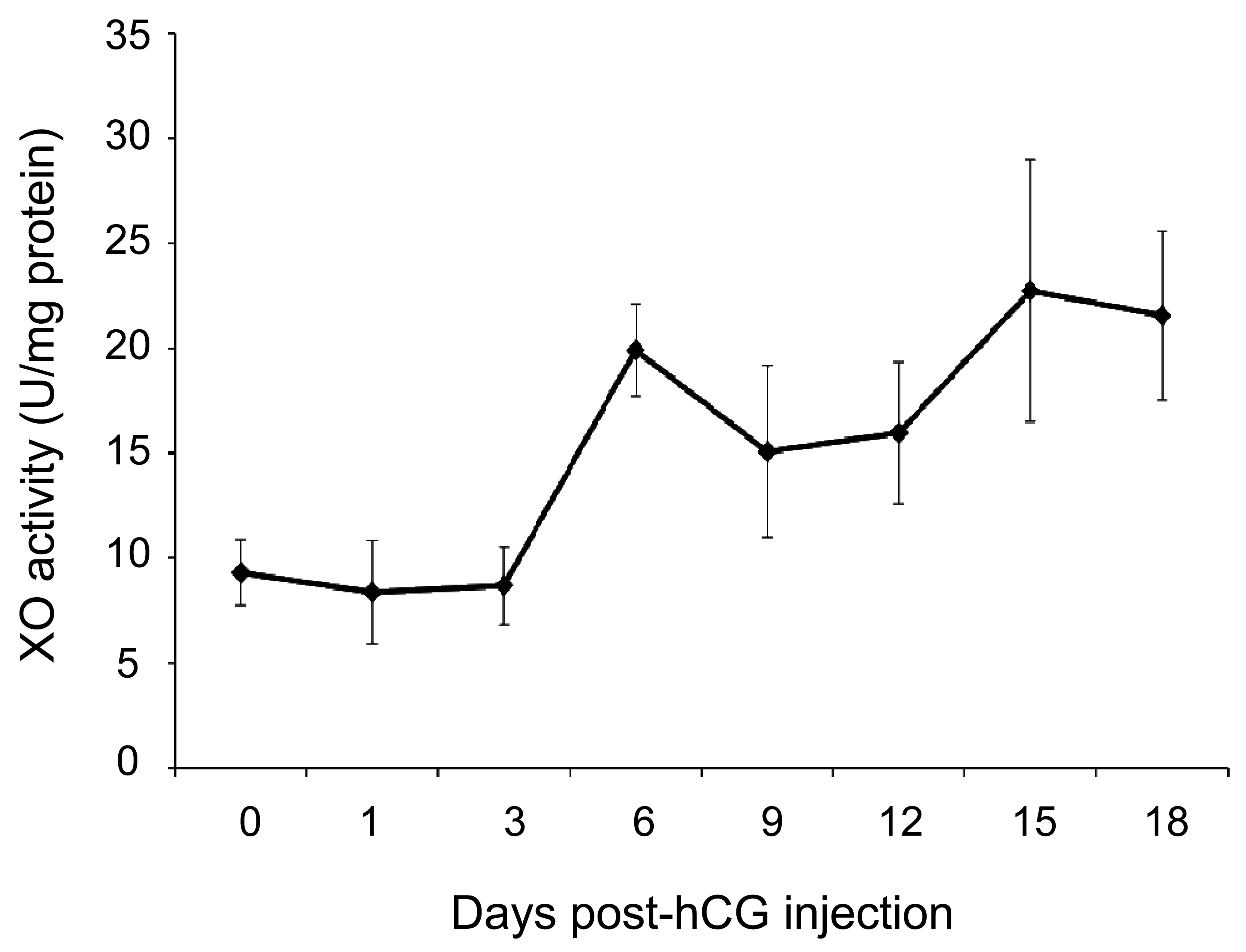

Supplement: Figure S1 — Xanthine oxidase (XO) activity can be detected in testis. Assay for XO was performed on testis of eels (n = 5 per group) at various days of post-hCG injection. (TIF) [file pone.0016938.s001.tif]

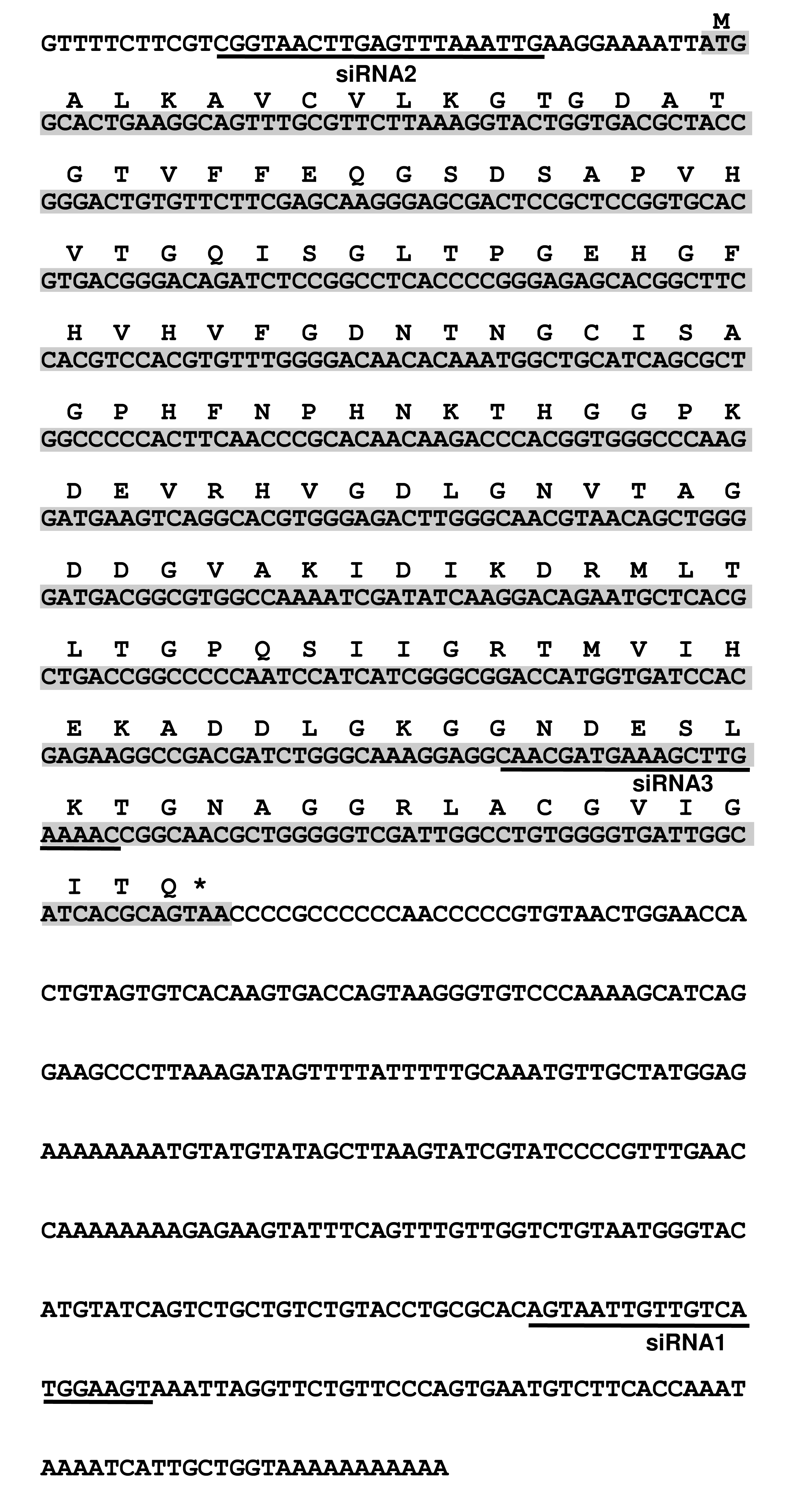

Supplement: Figure S2 — eCu/Zn SOD nucleotide sequence and siRNA target sites. Eel Cu/Zn SOD (eCu/Zn SOD) gene was obtained by cDNA library screening in eel testis. Gray shaded region indicates ORF region. Bold letters are deduced amino acids sequence. The three target sequences for the siRNA are underlined. *, stop codon. (TIF) [file pone.0016938.s002.tif]

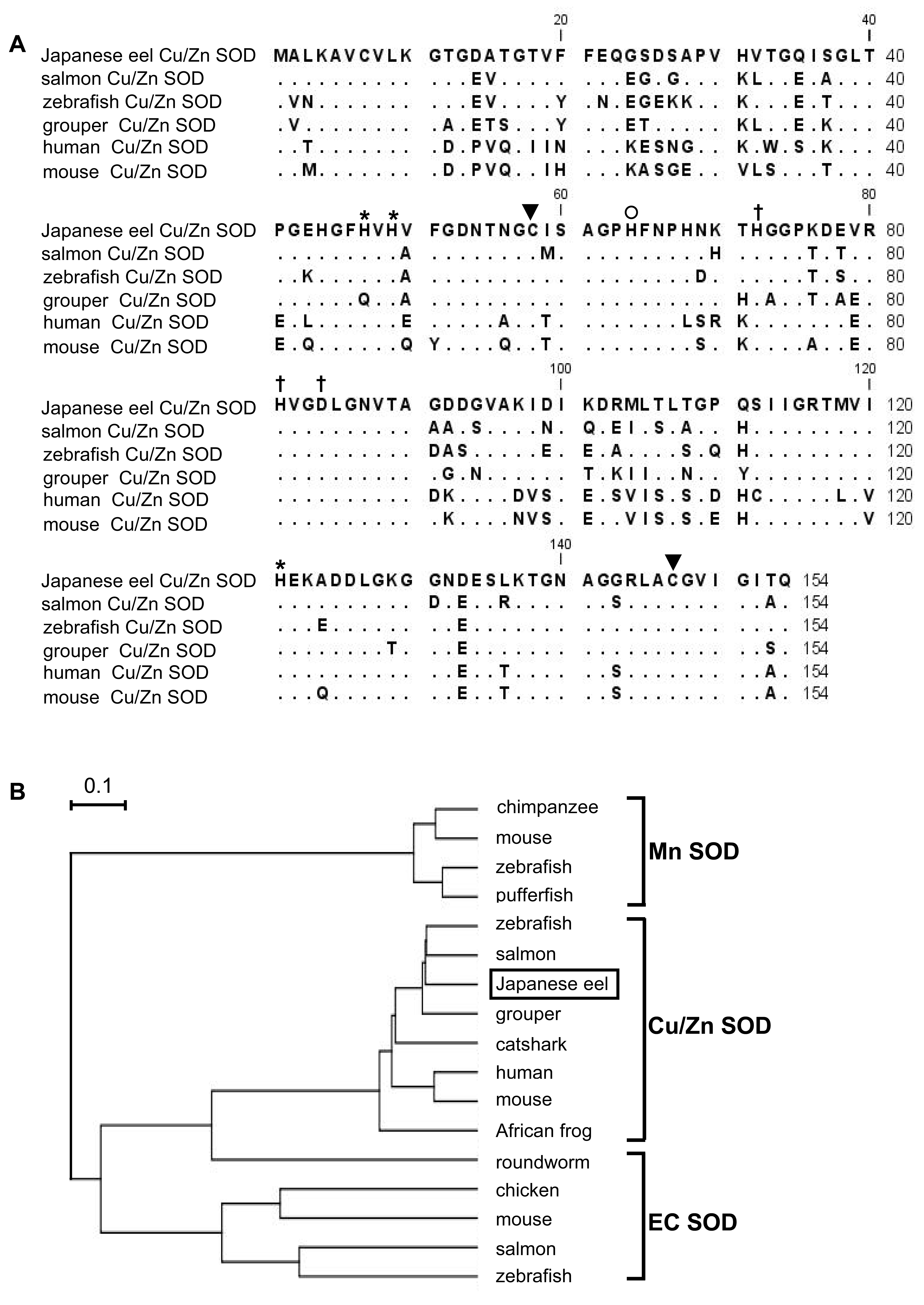

Supplement: Figure S3 — Deduced amino acid sequence, sequence alignment and phylogenetic analysis of eCu/Zn SOD . (A) Deduced amino acid sequence of eCu/Zn SOD and alignment with other species Cu/Zn SOD. Two cysteine residues are indicated by arrows. The copper (Cu) binding sites are indicated by asterisk and the zinc (Zn) binding sites are indicated by † sign. The common binding sites for Cu and Zn are indicated by an open circle. Dots are identical amino acids among the proteins. The eCu/Zn SOD amino acids sequence is highly homologous to salmon, Salmo salar (AY736282); zebrafish, Danio raerio (Y12236); grouper, Epinephelus coides (AY735008) (77.7–80.8%); human, Homo sapiens (AY450286) and mouse, Mus musculus (M35725) (67.6–71.7%), Cu/Zn SOD proteins. (B) Phylogenetic analysis of SOD proteins. Sequences were aligned using CLUSTAL W. Gene bank accession nos. for amino acid sequences are: chimpanzee (Pan troglodytes) Mn SOD (AB087274), mouse Mn SOD (X04972), zebrafish Mn SOD (AY195857), pufferfish, Takifugu obscurus, Mn SOD (EF667049), zebrafish Cu/Zn SOD, salmon CuZn SOD, grouper Cu/Zn SOD, catshark, Scyliorhinus torazame, Cu/Zn SOD (DQ988331), human Cu/Zn SOD, mouse Cu/Zn SOD, African frog, Xenopus laevis, Cu/Zn SOD (BC070696), roundworm, Caenorhabditis elegans, EC SOD (AB190513), chicken, Gallus gallus, EC SOD (BX929804), mouse EC SOD (AF223251), salmon EC SOD (BT046917) and zebrafish EC SOD (CT737206). Branch lengths indicate proportionality to the amino acid changes on the branch. Scale bar shows substitution per site. Mn SOD, manganese SOD; EC SOD, extracellular SOD. (TIF) [file pone.0016938.s003.tif]
