# Supplementary material for: Tolerance of Spermatogonia to Oxidative Stress Is Due to High Levels of Zn and Cu/Zn Superoxide Dismutase
Source: PLoS One. 2011 Feb 18;6(2):e16938. doi: 10.1371/journal.pone.0016938 (PMC3041797; doi:10.1371/journal.pone.0016938)
Supplement: Table S1 — Cu/Zn-SOD siRNA targeting sequences and Cu/Zn-SOD siRNA sequences. S, sense; A, antisense. (DOC) [file pone.0016938.s004.doc]

**Table S1.** Cu/Zn-SOD siRNA targeting sequences and Cu/Zn-SOD siRNA sequences.

| **siRNA name** | **Targeting sequence** | **siRNA sequence^a^** |
| --- | --- | --- |
| eCu/Zn SOD siRNA1 (position 749-769) | AGTAATTGTTGTCATGGAAGT | S: 5’-AGUAAUUGUUGUCAUGGAAGU-3’ |
|  |  | A: 5'-UUCCAUGACAACAAUUACUGU-3' |
| eCu/Zn SOD siRNA2 (position 9-29) | CGGTAACTTGAGTTTAAATTG | S: 5'-CGGUAACUUGAGUUUAAAUUG-3' |
|  |  | A: 5'-AUUUAAACUCAAGUUACCGAC-3' |
| eCu/Zn SOD siRNA3 (position 432-452) | CAACGATGAAAGCTTGAAAAC | S: 5'-CAACGAUGAAAGCUUGAAAAC-3' |
|  |  | A: 5'-UUUCAAGCUUUCAUCGUUGCC-3' |

^a^ S, sense; A, antisense.
